# Effects of the Problem-Oriented Learning Model on Middle School Students’ Computational Thinking Skills in a Python Course

**DOI:** 10.3389/fpsyg.2021.771221

**Published:** 2021-12-07

**Authors:** Hongquan Bai, Xin Wang, Li Zhao

**Affiliations:** School of Education Science, Nanjing Normal University, Nanjing, China

**Keywords:** computational thinking, problem-oriented learning model, programming teaching, Python, middle school students

## Abstract

The rapid development of computers and technology affects modern daily life. Individuals in the digital age need to develop computational thinking (CT) skills. Existing studies have shown that programming teaching is conducive to cultivating students’ CT, and various learning models have different effects on the cultivation of CT. This study proposed a problem-oriented learning (POL) model that is closely related to programming and computational thinking. In all, 60 eighth-grade students from a middle school in China were divided into an experimental group (EG) which adopted the POL model, and a control group (CG) which adopted the lecture-and-practice (LAP) learning model. The results showed that the students who were instructed using the POL model performed better than those who were instructed using the LAP model on CT concepts, CT practices, and CT perspectives. Significant differences were found for CT concepts and CT perspectives, but not for CT practices. Findings have implications for teachers who wish to apply new learning models to facilitate students’ CT skills, and the study provides a reference case for CT training and Python programming teaching.

## Introduction

The younger generation interacts frequently with technologies that permeate all aspects of their lives on a daily basis (Baruch and Erstad, 2018), and they are increasingly expected to be not only consumers but also producers of technology (Kong et al., 2020). In the digital age, computational thinking (CT) can develop students’ abilities of critical thinking, creative thinking, and problem solving (Ananiadou and Claro, 2009; Mishra and Yadav, 2013; Repenning et al., 2015). CT, just like reading, writing, and arithmetic, is a basic skill for all students (Wing, 2006). According to Bundy (2007), CT influences the research of almost all disciplines in natural science and human science. Many researchers consider that CT should be integrated into the formal education system as a learning objective to cultivate students’ ability to guide their future lives (Grover and Pea, 2013).

Recently CT related to programming has been included in K-12 courses around the world (Shute et al., 2017; Hsu et al., 2018; Sands et al., 2018). There is a consensus that students’ CT can be nurtured via programming education (Rich et al., 2017; Nouri et al., 2020). Most studies use visual programming tools, such as App Inventor and Scratch, which are closer to the representation of human language, helping students concentrate on the logic and structure, and become involved in programming instead of being anxious about the difficulties of writing programs (Kelleher and Pausch, 2005). However, few studies have used Python. Wen et al. (2014) pointed out that Python is suitable for cultivating students’ CT and problem-solving skills. Python has been found to be a means of helping learners develop skills to face real-world problems (Tang et al., 2020). Lee and Cho (2017) also mentioned that Python is a programming language that is of interest to beginners and is easy to learn. It is also used as an intermediate language for connecting modules written in other languages. At the same time, Lee and Cho (2017) found that teaching methods that use LAP learning, collaborative learning, game-based learning, and design-based learning environments are often used to cultivate students’ CT. LAP learning can help learners complete tasks in a limited amount of time, but this choice is a negative factor for students’ creativity. Pair programming is a form of collaborative learning. Studies have shown that pair programming has a positive impact on friend relationships, but has no effect on non-friend relationships (Werner et al., 2013). In game-based learning environments, the motivation to complete a game may adversely affect the learning of a certain level, thereby affecting learning from playing the game (Israel-Fishelson and Hershkovitz, 2019). Unplugged programming activities allow students to participate in computer science practice without using digital equipment, which can solve the limitations of computer hardware equipment and students’ lack of early programming knowledge, but this method is more suitable for the early stages of elementary education (Sun et al., 2021). Design-based learning (DBL) activities, such as interactive web design and digital storytelling, allow students to use multiple technologies to conduct activities, which imposes heavy curriculum burdens on teachers and adversely affects the entire process (Saritepeci, 2020). This study took a Python programming course as the context to construct a problem-oriented learning (POL) model to effectively promote students’ CT skills.

## Literature Review

### Computational Thinking

Computational thinking is a method of designing systems, solving problems, and understanding human behavior (Wing, 2006). It includes engineering and design thinking (effective solution developing), mathematical thinking to solve various problems, and system thinking (system understanding and modeling). Abstraction, decomposition, algorithms, and debugging are the CT components that most frequently arise in the literature (Shute et al., 2017). The International Society for Technology in Education (ISTE) defined CT as the common skills of algorithmic thinking, creativity, critical thinking, cooperative thinking, problem solving, and communication skills (ISTE, 2015). The goal of developing CT is not as a replacement for creative thinking, critical thinking or other kinds of thinking skills, but rather to increase the skills of using computers and algorithms to solve problems (Wing, 2011; Furber, 2012).

Computational thinking is a basic skill for all people (Wing, 2006), and will be used everywhere (Wing, 2008). Educational researchers have been actively seeking innovative methods and ways to incorporate CT into the curriculum and encourage students to participate in CT (Bower et al., 2017). They have attempted to teach and develop the knowledge and skills of CT in different educational situations by various means including programming (Basu et al., 2017; Bati, 2021), educational robotics (Chevalier et al., 2020; Qu and Fok, 2021), unplugged activities (Kuo and Hsu, 2020; Huang and Looi, 2021), games/simulations (Danial et al., 2021; Hooshyar et al., 2021), storytelling (Soleimani et al., 2019; Parsazadeh et al., 2020), and so forth. These tools become “technical partners in the learning process” (Jonassen et al., 2012), and the rationale for improving CT skills in each of these tools emphasizes various CT components (Shute et al., 2017). In addition, existing research has developed CT interventions in different disciplines such as physics and biology (Sengupta et al., 2013), expository writing and journalism (Wolz et al., 2011), mathematics (Wilkerson-Jerde, 2014), science in general (Weintrop et al., 2016; Basu et al., 2017), and science and arts (Sáez-López et al., 2016).

Previous studies, however, preferred to apply visual programming languages, while paying less attention to text programming languages. Nonetheless, visual programming is not as reliable as text programming, and its functions are not as good as the latter. Deng et al. (2020) pointed out that visual programming requires learners to focus on a great number of grammar rules, and the programming foundation is needed to develop CT. Thus, visual programming alone may not be sufficient for students to understand the true meaning of programming and to master CT, especially for beginners. Text programming can compensate for visual programming based on program functionality (Weintrop and Wilensky, 2015). In the era of artificial intelligence, Python has become the preferred development language for artificial intelligence applications (Okonkow and Ade-Ibijola, 2021). Compared with other text programming languages, Python is closer to human languages as it conforms to people’s thinking habits. It can reduce unnecessary grammar learning, thereby reducing cognitive load and allowing students to focus on solving programming problems (Maria and Tsiatsos, 2018). Kim et al. (2019) developed a data visualization education program, and the sixth-grade students received 6 days and 36 h of training. It was found that Python for data visualization education can effectively improve the CT of sixth graders, including their computational cognition, fluency, originality, and elaboration. Another study used the Python language to develop a learning program and model, and applied it to the 10-h learning of sixth-grade students. It was found that the robot-based Python learning model had a significant effect on improving students’ thinking skills, which confirms the applicability of the text-based programming language to elementary school students. Other studies (e.g., García Monsálvez, 2017; Lee and Cho, 2017; Maria and Tsiatsos, 2018) also found that Python programming education had a positive impact on students’ CT.

At present, there is no common definition of CT. Therefore, the evaluation methods of CT are very diverse. A CT questionnaire based on the five CT factors proposed by ISTE was designed by Durak and Saritepeci (2018), namely algorithmic thinking, creativity, problem solving, cooperation, critical thinking, and communication. Brennan and Resnick (2012) proposed a three-dimensional CT framework for visual programming using Scratch, and pointed out that the framework could be transferred to other programming teaching practices such as Logo programming (Lye and Koh, 2014). The framework consists of three dimensions: CT concepts (the concepts which designers use when they are programming), CT practices (the practices that designers develop while they are programming), and CT perspectives (the perspectives on the world and themselves that designers form).

### Learning Models of Programming Teaching

An increasing amount of attention is being paid to programming teaching. Garneli et al. (2015) pointed out that game design, robotics teaching, project-based learning, and collaborative learning are becoming increasingly popular in programming teaching. The teaching intervention of Vihavainen et al. (2014) included collaboration and peer support. Florez et al. (2017) further pointed out the importance of using visualization tools to help students develop programming concepts. Scherer et al. (2020) also found from meta-analysis that visualization had a moderate effect on programming learning, whereas physicality had a large effect.

Many teaching methods have been explored and applied to improve students’ programming skills. For example, Olelewe and Agomuo (2016) discussed the influence of two teaching methods on programming learning. The results showed that the B-learning model (the combination of e-learning and traditional face to face learning) could improve students’ programming language performance more effectively than the traditional face-to-face model. Researchers have found that pair programming can improve personal programming skills, programming efficiency and quality (Zhong et al., 2017), while also increasing self-confidence in learning (Lai and Xin, 2011). Corral et al. (2014) found that game-oriented methods based on interaction with tangible user interfaces could improve students’ motivation and academic performance. Uysal (2014) indicated that problem-solving instructional methods can effectively improve students’ academic performance and problem perception. Early studies adopted the problem-solving approach to give students the detailed steps and sequences of behaviors to solve the problems they encountered while they were coding (Scherer et al., 2020).

Scherer et al. (2020) conducted a meta-analysis of some instructional approaches and found that blended learning (1.023) had the largest intervention effect, followed by game-based learning (0.821) and metacognitive strategies (0.658), and finally collaborative activities (0.560), problem solving instruction (0.518), and feedback strategies (0.436). At the same time, the effectiveness of teaching methods may vary depending on the research content and teaching conditions (Li and Ma, 2010), and an integration of various teaching methods should be more effective for tutoring programming (Vihavainen et al., 2014).

In recent decades, the study of K-12 programming teaching was mostly carried out in high schools. For example, He et al. (2014) studied robotics programming teaching of collaboration with a robotics club for high school students. However, scanty attention has been paid to investigating the model of programming teaching for middle school students. Children should start to learn programming at a much younger age to motivate their learning interest. This study describes a Python programming course for eighth graders. Although there are many innovative teaching methods, they have not been applied to specific programming courses and cannot guide programming teachers’ teaching well. Therefore, teachers often use LAP methods in the programming classroom (Kim and Yun, 2020). Teachers demonstrate the correct steps, and then students imitate to complete the task, which is not conducive to the cultivation of students’ problem-solving ability and computational thinking. Therefore, this study proposed the POL model to promote eighth graders’ CT.

### The Problem-Oriented Learning Model

Polya (1957) proposed a four-step process to solve problems; this process is widely used around the world to help people with problem solving: understand the problem, devise a plan, carry out the plan, and look back. In other words, problem solving covers a series of processes. On this basis, Polya, Beichner (2002) developed GOAL-oriented problem solving for physics, which involves collecting information about the problem, figuring out an approach to the problem, analyzing the problem, and learning from one’s efforts. Evidence collected showed that this approach had a positive impact on students’ ability to solve problems. Kalelioğlu et al. (2016) proposed a framework of CT as a process of problem-solving, including identifying the problem; collecting, representing, and analyzing data; generating, choosing, and planning solutions; implementing solutions; and evaluating solutions and continuing for improvement. Kim and Yun (2020) proposed a learning model focused on CT skills, including problem identifying, analyzing, systematizing, and solving. These studies show that problem solving covers a series of processes which differ in specific teaching situations.

In the literature review, many problem-solving instruction approaches have been created in the fields of mathematics (e.g., Suarsana et al., 2019), science (e.g., Akben, 2020), and physics (e.g., Dewi et al., 2019), but few have been applied to Python courses. Most of the research has been to improve problem-solving skills (e.g., Cheng et al., 2018), but the impact on CT is still unclear. In fact, CT, programming skills, and problem solving are closely connected. Kalelioğlu et al. (2016) found that “abstraction,” “problem,” and “solving” are the most commonly used words in the definition of CT. Román-González et al. (2017) developed a scale to measure CT, and the results showed significant correlations with problem-solving skills. Based on the studies of Polya (1957), Beichner (2002), Kalelioğlu et al. (2016), and Kim and Yun (2020), this study formed a POL model including problem decomposition, problem abstraction, algorithmic representation, solution evaluation, and generalization and migration. Compared with previous studies, the framework of the POL model in this study designed five specific phases to solve programming problems, and it was more suitable for Python programming courses. Therefore, this study applied the POL model to Python programming courses to promote the development of students’ CT.

### Research Questions

Based on the needs of CT training and its close relationship with programming and problem solving, this study focused on a middle school Python programming course to construct a POL model oriented toward programming problems that effectively cultivates students’ CT. Furthermore, it explored the effectiveness of the POL model for the cultivation of CT through experiments.

The questions this study aimed to address are: Compared with the LAP model, did the POL model significantly improve students’ CT concepts, CT practices, and CT perspectives?

## Materials and Methods

### Participants

A total of 60 eighth graders from a middle school in China participated in the study. In this school, the learning competence of students in different grade 8 classes is similar, due to the school having implemented the parallel classes method according to the students’ previous academic performance when allocating students to classes. We randomly selected two classes taught by the same teacher to participate in the study. One class was assigned as an experimental group (EG) and another as a control group (CG). There were 30 students with 17 males and 13 females in the EG, who received the guidance of the POL model. The CG was composed of 30 students with 15 males and 15 females, who learned via LAP learning model. Students in both groups had already taken a one-semester Python programming course. The results of the Python final test of the previous semester showed no significant difference between the two groups (*t* = 1.241, *p* = 0.220 > 0.05). It could therefore be considered that the two groups had the same initial learning level.

Participants in this study were involved on a voluntary basis and with the approval of their parents. To protect the participants, their personal information was hidden during the study. In addition, they could withdraw from the study at any time.

### Instructional Design

#### Selection of Instructional Content

The course of Python programming covers a wide range of content, including basic input and output, branch selection, loops, and so on, some of which had been taught in the previous semester. This study selected the following three units as the instructional content: list, string and dictionary, and custom function (see Table 1).

**TABLE 1:** Programming issues corresponding to the unit.

Unit	Programming problem
List	Number of daffodils, statistics, average age, etc.
String and dictionary	Compress, decipher mail, exchange parity, etc.
Custom function	Perfect numbers between positive integers 2 and N, number of primes, palindrome three prime numbers, etc.

#### The Problem-Oriented Learning Model

**FIGURE 1:**
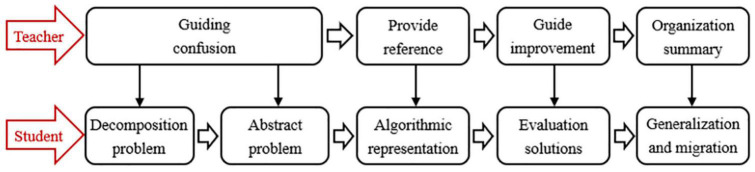
Problem-oriented learning model.

Combining the descriptions of the problem-solving process in related research (Kalelioğlu et al., 2016; Kim and Yun, 2020), this study divided the POL model into the following processes, as shown in Figure 1.

Student: First, problem decomposition. Decompose complex problems, extract the key information in the problem, and transform it into a problem that the student knows how to solve. Second, problem abstraction. Use digital language to express text information, abstract actual problems into mathematical problems and model them. Third, algorithmic representation. Express the logic of solving the problem in the Python programming language. Finally, solution evaluation. Use the Python programming language to test the effectiveness of the algorithm; evaluate and optimize the algorithm during continuous debugging. Step 5: generalization and migration. Generalize the solution of the problem and migrate it to other similar programming problem solutions.

Teacher: First, guide students to decompose questions and find out the key information in the problem. Second, guide students to convert text information into mathematical models. Third, provide a programming reference module when students express problem solutions with algorithms, and guide students to debug and optimize the algorithms. Finally, summarize the problem-solving process and give similar problems to promote the development of students’ transfer ability.

#### The Lecture-and-Practice Learning Model

**FIGURE 2:**
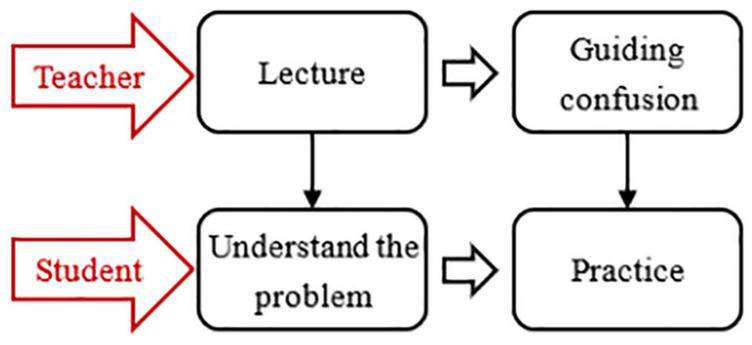
Lecture-and-practice learning model.

The CG adopted the LAP learning model, as shown in Figure 2. Teacher: First, teach the problem-solving process, and then give timely guidance when students practice by themselves. Student: Listen carefully and record when the teacher teaches, and then practice according to the teacher’s steps.

#### The Difference Between the Two Learning Models

The LAP model is often used in programming teaching, whereas this study adopted the POL model in the Python programming course. The two mainly differ in terms of the teaching process and the behavior of teachers and students, as shown in Table 2. Take the “number of daffodils” as an example. In the LAP class, the teacher first analyzes the problem and explains it. For example, what is the number of daffodils? How can we find the number of daffodils? How can we implement it in Python? In the course of the explanation, the code is written gradually (Understand the problem). After the teacher explains, the students refer to the teacher’s code to solve the problem (Practice). When the students are confused, the teacher will guide or explain in groups. In contrast, in the POL class, the teacher first guides students to think about the problem to be solved (Decomposition problem), then guides them to think about the characteristics of daffodil numbers and how to find the ones, tens, and hundreds of a number (Abstract problem). Students try to implement it in Python. If they encounter difficulties, the teacher can prompt them appropriately. For example, the teacher can prompt the students to use the remainder operation (Algorithmic representation). After students finish writing the code, they will debug and think about how to optimize the algorithm (Evaluation solutions). Finally, the teacher gives a similar question, such as finding the number of lightning strikes, to promote students’ summary and migration (Generalization and migration).

**TABLE 2:** The difference between the two learning models.

Learning models	Teaching process	Teacher activities	Student activities
POL model	Teachers guide the whole process; students solve the problem independently	1. Guiding confusion 2. Provide reference 3. Guide improvement 4. Organization summary	1. Decomposition problem 2. Abstract problem 3. Algorithmic representation 4. Evaluation of solutions 5. Generalization and migration
LAP learning model	Students imitate after the teacher demonstrates	1. Analyze the problem 2. Teaching process 3. Guiding confusion	1. Understand the problem 2. Practice

### Procedure

The experimental school has its own self-developed online program evaluation system. The platform integrates the Python language compiler and the programming question library. In addition, the platform provides online test and evaluation functions. After the students submitted the questions, the platform immediately gave feedback, including the scores obtained and the errors, which provided the students with the opportunity to continuously debug.

The experiment lasted for one semester, from February 2019 to June 2019, a total of 22 weeks, each of which was 40 min. Three units were taught during the semester. Before the instruction, the two groups completed the pre-test of CT perspectives scale. At the end of each of the three units, the corresponding unit test was carried out. During the learning activity, the students in the EG adopted the POL model and the CG adopted the LAP learning model. After completing the learning tasks of the three units, the students took the post-test of the CT perspectives scale, the Bebras test, and the computer-based final test. The experimental procedure is shown in Table 3.

**TABLE 3:** Experimental procedure.

	EG (*n* = 30) POL model	CG (*n* = 30) LAP learning model
1 week	Pre-test of CT perspectives scale	
6 weeks	Unit 1: List	
1 week	Unit 1 test	
4 weeks	Unit 2: String and dictionary	
1 week	Unit 2 test	
4 weeks	Unit 3: Custom functions	
1 week	Unit 3 test	
4 weeks	Computer-based final test + Bebras test + post-test of CT perspectives scale	

### Measurement

Some researchers have incorporated Brennan and Resnick’s (2012) framework into the evaluation of CT (Lye and Koh, 2014; Grover et al., 2015; Kong, 2019; Mouza et al., 2020). Combined with the teaching content and the characteristics of the Python programming course, this study modified their framework and formulated the CT evaluation for this study. Although many efforts have been made regarding CT evaluation (Basawapatna et al., 2011), it is still a challenge to evaluate CT learning in a programming environment. Survey with questionnaires is the most common way to measure CT attitudes or knowledge (e.g., Kim et al., 2013; Jun et al., 2014). Reflection is also often used in studies where students are asked to reflect on their programming experience (e.g., Zimmerman and Tsikalas, 2005; Yang, 2010). Other studies have tested the dimension of subject knowledge in learning achievement after integrating CT with disciplinary teaching (e.g., Sengupta et al., 2013). This study used the tools in Table 4 to measure the three dimensions of CT.

**TABLE 4:** Measurement tools.

Measurement dimensions	Measurement tools
CT concepts	Unit test (single-choice questions and program comprehension questions)
	Computer-based final test
CT practices	Unit test (program correction questions)
	Bebras test
CT perspectives	CT perspectives scale

#### Computational Thinking Concepts

Brennan and Resnick (2012) defined CT concepts as including sequences, loops, parallelism, events, conditionals, operators, and data. Considering that this study was carried out in the context of a Python programming course, the two concepts of sequences and parallelism were not included in the CT concepts. In addition, the concept of events is rarely mentioned in Python programming, but the concept of functions is commonly used. Therefore, functions were used instead of events in this study (see Table 5).

**TABLE 5:** Common CT concepts in Python.

CT concepts	Implication
Operators	Operators provide support for mathematical, logical, and string expressions. In Python, there are arithmetic operators (addition, subtraction, multiplication, division, etc.), relational operators (equal, less than, etc.), logical operators (and, or, etc.), etc.
Data	Data involve storing, retrieving, and updating values. In Python, strings, lists, dictionaries, etc. are all sequences used for data storage.
Conditionals	A conditional is a code block that judges whether to execute through the result of one or more statements (True or False). In Python, it often corresponds to the use of “if-elif-else” statements.
Loops	A loop is a mechanism for running the same instruction multiple times. In Python, it often corresponds to the use of “for loops” and “while loops.”
Functions	Functions are organized and reusable. Code segments are used to implement single or related functions. In Python, it often corresponds to the use of built-in functions and custom functions.

Single choice questions and program comprehension questions in the unit test and computer-based final test were used to analyze CT concepts. Single choice questions and program comprehension questions in the unit test reflect the understanding of CT concepts, and the final test based on the computer reflects the application of CT concepts. This study carried out three unit tests which were prepared by the researcher and the teacher, and were distributed at the end of each unit. Well-designed choice measurements could be applied to further learners’ understanding (Glass and Sinha, 2013) and provided them with feedback and explanations (Black and Wiliam, 1998). The computer-based final test was selected by the researchers and the teacher and was distributed at the end of the semester.

#### Computational Thinking Practices

Computational thinking practices are iterative and incremental, and include testing and debugging, reusing and remixing, and abstracting and modularizing according to Brennan and Resnick (2012). Considering that this study was set in the context of a Python course and did not involve the production and completion of complex products or huge projects, the CT practices of this study only included testing, debugging, reusing, and remixing. Testing and debugging are to ensure that the program can run automatically and efficiently. They are indispensable practices in programming activities. Testing is to find errors, and debugging is to correct errors. Reusing and remixing are based on the problem solutions given by the samples to construct the solutions that include learners’ own ideas. Reusing and remixing are also a process of summarizing problem solutions and migrating to other related problems.

Program correction questions in the unit test and Bebras test were used to analyze CT practices. Among them, the first was the evaluation of the ability of “testing and debugging” in CT practices, and the latter was the evaluation of the ability of “reusing and remixing” in CT practices. The program correction questions were designed to improve the learners’ ability to read and understand the code (Lopez et al., 2008). The Bebras test, an international challenge in informatics and CT, aims to improve and cultivate the CT ability of primary and secondary school students (about 8–18 years old). Bebras reflects the contestants’ CT ability through real-life problems and some focus issues. The Bebras test questions in this study were selected from the Bebras tests in 2016 and 2017.

#### Computational Thinking Perspectives

Computational thinking perspectives include expressing (computational thinkers see computation as something they can use to design and self-express), connecting (recognizing the importance of creating with others and the value of creating for others), and questioning (feeling empowered to ask questions about and with technology) based on Brennan and Resnick (2012). ISTE and the Computer Science Teachers Association (CSTA) considered CT as a problem-solving process that includes the following dispositions or attitudes: confidence in dealing with complex task, persistence in solving the difficult task, tolerance for ambiguity, the ability to deal with open-ended problems, and the ability to cooperate with others (ISTE and CSTA, 2011). The framework of the Hong Kong CoolThink@JC Jockey Club’s CT education curriculum includes self-expression, questioning and understanding, connecting with life, digital capability, and computational identity.

The CT perspectives in this study consist of four aspects: creation and expression, communication and cooperation, questioning, and problem solving. According to the ISTE definition of CT, Korkmaz et al. (2017) designed a computational thinking scale (CTS) to evaluate CT in algorithmic thinking, creativity, cooperativity, critical thinking, and problem solving. This study refers to CTS and selected items related to CT perspectives to determine the CT perspectives scale, as shown in Table 6. There are 11 items rated using a 5-point Likert scale (from 1 – strongly disagree, to 5 – strongly agree) in the scale.

**TABLE 6:** CT perspectives scale.

CT perspectives	Items
Creation and expression	I am happy to use the computing tools around me (computer hardware and software) to create and express my ideas.
	Learning to use programming languages (Python, C++, etc.) to solve problems encountered makes me feel very proud.
Communication and cooperation	Rather than doing it independently, I prefer to communicate with classmates or teachers about problems encountered and I am willing to work together to solve them.
	I think that in the process of exchanges and cooperation, I can generate more ideas and gain more benefits.
Questioning	When I see new technologies such as face recognition and robotics, I often think about how they work.
Problem solving	I believe that I can solve most of the problems I encountered if I have enough time and effort.
	When there is a problem, I will keep thinking over the problem without proceeding to another subject.
	I believe that I am able to solve the problems that might occur when I encounter a new situation.
	I trust my intuitions and feelings of “trueness” and “wrongness” when I carry out the solution of a problem.
	It is interesting to try to solve complicated problems.
	I like to learn things with challenge.

To measure the validity and reliability of the CT perspectives scale, two classes were selected for trial testing before the formal experiment, and finally 54 valid data were collected. In the study, the Cronbach’s α value was 0.962, showing acceptable reliability in internal consistency. Kaiser–Meyer–Olkin (KMO, =0.854) and Bartlett’s test (*p* = 0.00 < 0.05) were calculated to test the validity of the scale.

### Data Analysis

In the study, SPSS 22.0 was used to analyze the qualitative and quantitative data collected during the experiment. Single choice questions and program comprehension questions in the unit test and computer-based final test were used to evaluate the CT concepts. Program correction questions in the unit test and the Bebras test were used to evaluate the CT practices. The CT perspectives scale was used to evaluate CT perspectives. For the unit test, Bebras test, and CT perspectives scale, the independent sample *t*-test were applied. Analyzing the computer-based final test was to extract the application times of CT concepts in the code, and then an independent sample *t*-test was conducted.

## Results

This study conducted statistical analysis of the collected qualitative and quantitative data from CT concepts, CT practices, and CT perspectives. According to the normality test, the results of the Unit Test, Computer-based Final Test, Bebras Test, and CT Perspectives Test all conformed to normal distribution (*p* = 0.200* > 0.05). Thus, the independent sample *t*-test was used to test the difference between groups and the pre- and post-test. Cohen’s *d*, which is widely used for the standardization effect in the *t*-test (Cuthill et al., 2007), is the difference between the mean values of two groups divided by the standard deviation (equation 1). It is used to calculate the effects by comparing the mean values of two groups (Sullivan and Feinn, 2012). When the value of Cohen’s *d* is ≥ 0.2 and <0.5, it indicates a small effect. If the value is ≥0.5 and <0.8, it shows a moderate effect. When the value is ≥0.8, it means a large effect (Cohen, 1988). Cohen’s *d* was used as the auxiliary value of the *t*-test, where high efficiency should indicate a high experimental effect.

### Computational Thinking Concepts

#### Analysis of Single-Choice and Program Comprehension in the Unit Test

The content of the three units were list, string, and dictionary, and the custom function. The single-choice and program comprehension in the unit test examined the students’ understanding of CT concepts. The total scores of the three units were 18, 26, and 9. The independent sample *t*-test results showed that the mean scores of single-choice questions and program comprehension questions in the unit tests of the EG were higher than those of the CG (see Table 7). The results showed a significant difference in the “list” unit (*t* = 3.38, *p* = 0.001 < 0.01, *d* = 0.88), the “string and dictionary” unit (*t* = 2.01, *p* = 0.049 < 0.05, *d* = 0.53), and the “custom function” unit (*t* = 2.50, *p* = 0.015 < 0.05, *d* = 0.66). The Cohen’s *d* was 0.88, 0.52, and 0.66, indicating that the POL model had a large effect on students’ “list” unit learning, and a medium effect on their “string and dictionary” unit and “custom function” unit learning. In terms of the dispersion degree of data, the standard deviation of CG (8.69) was much larger than that of EG (5.75), indicating that the CG sample data had greater volatility and their understanding of CT concepts was more unstable, while EG had a more accurate understanding of the concepts of CT.

**TABLE 7:** The independent sample *t*-test of single-choice and program comprehension in the unit tests of the two groups.

Unit	Group	*N*	*M*	*SD*	*t*	*p*	*d*
List	EG	30	14.27	3.265566	3.38	0.001**	0.88
	CG	30	11.17	3.83			
String and dictionary	EG	30	22.97	3.68	2.01	0.049*	0.53
	CG	30	20.43	5.82			
Custom function	EG	30	7.50	0.94	2.50	0.015*	0.66
	CG	30	6.83	1.11			
Total	EG	30	44.73	5.75	3.12	0.003**	0.87
	CG	30	38.43	8.69			

*p < 0.05, **p < 0.01.

#### Analysis of the Computer-Based Final Test

By analyzing the code in the computer-based final test, the qualitative code data were converted into quantitative data. The qualitative data were the students’ code, and the quantitative data were the number of code blocks that reflect each CT concept in the code. For example, if “if-else” appeared twice in the student’s code, then the CT concept “Conditionals” was increased twice; if there were two “while loops” and one “for loop” in the student’s code, the CT concept “Loops” would increase three times; if students used a custom function and a built-in function in their code, the CT concept “Functions” was increased twice. The number of applications of each CT concept was extracted from the code. The more application of the CT concepts, the more familiar the students were with this concept. The computer-based final test examined the students’ application of CT concepts. The independent sample *t*-test results of the computer-based final test are shown in Table 8. The mean application of operators, conditionals, data, loops, and functions in the EG was higher than that in the CG. The results showed a significant difference among the application of CT concepts of operators (*t* = 2.35, *p* = 0.029 < 0.05, *d* = 1.02), conditionals (*t* = 2.31, *p* = 0.030 < 0.05, *d* = 0.94), data (*t* = 2.08, *p* = 0.048 < 0.05, *d* = 0.85), and loops (*t* = 2.17, *p* = 0.041 < 0.05, *d* = 0.88), but there was no significant difference in functions (*t* = 1.13, *p* = 0.269 > 0.05, *d* = 0.46). In general, there was a significant difference in the application of CT concepts between the two groups (*t* = 4.99, *p* = 0.000 < 0.001, *d* = 2.00). The Cohen’s *d* was greater than 0.8. It proved the POL model had a large effect on students’ application of CT concepts.

**TABLE 8:** The independent sample *t*-test on the two groups’ computer-based final test.

Unit	Group	*N*	*Max*	*M*	*SD*	*t*	*p*	*d*
Operators	EG	30	4	3.62	0.65	2.35	0.029*	1.02
	CG	30	4	2.85	0.99			
Conditionals	EG	30	6	4.85	0.69	2.31	0.030*	0.94
	CG	30	5	4.31	0.48			
Data	EG	30	6	3.77	0.83	2.08	0.048*	0.85
	CG	30	4	3.08	0.86			
Loops	EG	30	8	7.08	0.76	2.17	0.041*	0.88
	CG	30	8	6.31	1.03			
Functions	EG	30	4	2.23	0.93	1.13	0.269	0.46
	CG	30	3	1.85	0.80			
Total	EG	30	23	21.54	1.51	4.99	0.000***	2.00
	CG	30	21	18.38	1.71			

*p < 0.05, ***p < 0.001.

### Computational Thinking Practices

The CT practices in this study included “testing and debugging” and “reusing and remixing” practices. The program correction questions in the unit test examined the students’ “testing and debugging” abilities, and the total scores of the three units were 3, 5, and 2, respectively. The Bebras test examined the students’ ability to “reuse and remix,” with total scores of 36 and 54 for simple questions and difficult questions, respectively.

#### Analysis of Program Correction in the Unit Test

The independent sample *t*-tests were performed (see Table 9). The results showed that the mean scores of the program correction questions of students in the EG were higher than those in the CG in the list (*t* = 1.29, *p* = 0.203 > 0.05), string and dictionary (*t* = 0.63, *p* = 0.530 > 0.05), and custom functions (*t* = 0.81, *p* = 0.422 > 0.05), but there was no significant difference.

**TABLE 9:** The independent sample *t*-test of the two groups’ program correction questions in the unit test.

Unit	Group	*N*	*M*	*SD*	*t*	*p*	*d*
List	EG	30	2.60	1.04	1.29	0.203	0.35
	CG	30	2.20	1.35			
String and dictionary	EG	30	2.87	1.68	0.63	0.530	0.17
	CG	30	2.60	1.59			
Custom functions	EG	30	0.83	0.99	0.81	0.422	0.21
	CG	30	0.63	0.93			
Total	EG	30	6.03	2.23	1.73	0.089	0.45
	CG	30	5.40	1.77			

#### Analysis of the Bebras Test

The Bebras test can reflect students’ ability to transfer CT, and the premise of transfer is the ability to “reuse and remix.” The higher the ability to “reuse and remix,” the more students can transfer CT to the solution of related practical problems. The independent sample *t*-test result is shown in Table 10. It was found that the Bebras test score of the EG was higher than that of the CG, especially for difficult questions. No significant difference was found between the two groups for either simple questions (*t* = 1.14, *p* = 0.260) or difficult questions (*t* = 1.40, *p* = 0.166).

**TABLE 10:** The independent sample *t*-test of the two groups’ Bebras test.

Unit	Group	*N*	*M*	*SD*	*t*	*p*	*d*
Simple questions	EG	30	31.60	5.44	1.14	0.260	0.30
	CG	30	30.00	5.46			
Difficult questions	EG	30	42.27	9.11	1.40	0.166	0.37
	CG	30	38.87	9.67			
Total	EG	30	73.87	11.14	1.77	0.082	0.46
	CG	30	68.87	10.75			

### Computational Thinking Perspectives

The CT perspectives in this study consist of four aspects: creation and expression, communication and cooperation, questioning, and problem solving. To perform the independent sample *t*-test of the pre-test of the CT perspectives scale, the results showed no significant difference in creation and expression (*t* = 1.55, *p* = 0.126 > 0.05), communication and cooperation (*t* = 1.56, *p* = 0.125 > 0.05), questioning (*t* = 1.03, *p* = 0.305 > 0.05), or problem solving (*t* = 1.12, *p* = 0.268 > 0.05) between the two groups (see Table 11), indicating that the students in the EG were at the same level as the students in the CG before the experiment.

**TABLE 11:** The independent sample *t*-test of the two groups’ CT perspectives pre-test.

CT perspectives	Group	*N*	*M*	*SD*	*t*	*p*
Creation and expression	EG	30	7.87	1.57	1.55	0.126
	CG	30	7.20	1.75		
Communication and cooperation	EG	30	8.03	1.61	1.56	0.125
	CG	30	7.30	2.02		
Questioning	EG	30	4.00	1.05	1.03	0.305
	CG	30	3.73	0.94		
Problem solving	EG	30	23.70	2.84	1.12	0.268
	CG	30	22.70	3.99		
Total	EG	30	43.60	5.54	1.60	0.116
	CG	30	40.93	7.29		

The result of the independent sample *t*-test on the post-test of the CT perspectives scale is shown in Table 12. It was found that the mean scores of creation and expression (*t* = 2.03, *p* = 0.047 < 0.05), communication and cooperation (*t* = 2.89, *p* = 0.005 < 0.01), questioning (*t* = 2.77, *p* = 0.008 < 0.01), and problem solving (*t* = 2.35, *p* = 0.022 < 0.05) of the EG were higher than those of the CG, and there were significant differences. The Cohen’s *d* was 0.53, 0.73, 0.70, and 0.60, respectively, indicating that the POL model had a medium effect on students’ creation and expression, communication and cooperation, questioning, and problem solving.

**TABLE 12:** The independent sample *t*-test of the two groups’ CT perspectives post-test.

CT perspectives	Group	*N*	*M*	*SD*	*t*	*p*	*d*
Creation and expression	EG	30	8.73	1.78	2.03	0.047*	0.53
	CG	30	7.80	1.79			
Communication and cooperation	EG	30	8.80	1.32	2.89	0.005**	0.73
	CG	30	7.67	1.69			
Questioning	EG	30	4.47	0.78	2.77	0.008**	0.70
	CG	30	3.87	0.90			
Problem solving	EG	30	25.90	4.46	2.35	0.022*	0.60
	CG	30	23.50	3.38			
Total	EG	30	47.90	7.53	2.79	0.007**	0.70
	CG	30	42.83	6.52			

*p < 0.05, **p < 0.01.

The results of the paired sample *t*-tests on the two groups’ CT perspectives pre- and post-test are shown in Tables 13, 14. Although it was found that the mean scores of creation and expression (*t* = 1.31, *p* = 0.194 > 0.05), communication and cooperation (*t* = 0.76, *p* = 0.449 > 0.05), questioning (*t* = 0.56, *p* = 0.578 > 0.05), problem solving (*t* = 0.84, *p* = 0.405 > 0.05), and total scale (*t* = 1.06, *p* = 0.292 > 0.05) of the post-test were higher than those of the pre-test, there were no significant differences. However, it was found in the EG that the mean scores of creation and expression (*t* = 2.00, *p* = 0.050 = 0.05), communication and cooperation (*t* = 2.02, *p* = 0.049 < 0.05), problem solving (*t* = 2.28, *p* = 0.027 < 0.05), and total scale (*t* = 2.52, *p* = 0.015 < 0.05) of the post-test were higher than those of the pre-test, and there were significant differences. The Cohen’s *d* was 0.541, 0.532, 0.588, and 0.650, respectively, indicating that although the Python course cannot significantly improve students’ computational thinking, the Python course taught using the POL model can significantly improve students’ computational thinking.

**TABLE 13:** The paired sample *t*-test of the CG’s CT perspectives pre- and post-test.

CT perspectives	Group	*N*	*M*	*SD*	*t*	*p*
Creation and expression	Pre-test	30	7.20	1.75	1.31	0.194
	Post-test	30	7.80	1.79		
Communication and cooperation	Pre-test	30	7.30	2.02	0.76	0.449
	Post-test	30	7.67	1.69		
Questioning	Pre-test	30	3.73	0.94	0.56	0.578
	Post-test	30	3.87	0.90		
Problem solving	Pre-test	30	22.70	3.98	0.84	0.405
	Post-test	30	23.50	3.38		
Total	Pre-test	30	40.93	7.29	1.06	0.292
	Post-test	30	42.83	6.52		

**TABLE 14:** The paired sample *t*-test of the EG’s CT perspectives pre-test and post-test.

CT perspectives	Group	*N*	*M*	*SD*	*t*	*p*	*d*
Creation and expression	Pre-test	30	7.87	1.57	2.00	0.050*	0.541
	Post-test	30	8.73	1.78			
Communication and cooperation	Pre-test	30	8.03	1.61	2.02	0.049*	0.523
	Post-test	30	8.80	1.32			
Questioning	Pre-test	30	4.00	1.05	1.96	0.055	
	Post-test	30	4.47	0.78			
Problem solving	Pre-test	30	23.70	2.84	2.28	0.027*	0.588
	Post-test	30	25.90	4.46			
Total	Pre-test	30	43.60	5.54	2.52	0.015*	0.650
	Post-test	30	47.90	7.53			

*p < 0.05.

## Discussion

This study combined the process of solving programming problems to construct a POL model, and verified its effectiveness for CT training through the implementation of Python programming teaching.

### Computational Thinking Concepts

The evaluation of the CT concepts in this study included two aspects: understanding of the CT concepts and application of the CT concepts. Application of the CT concepts refers to the number of CT concepts included in the students’ programming works. According to the results, the understanding and application of the CT concepts by the students learning with the POL model were better than those of the students learning with the LAP model, and the difference was significant. This implied that the proposed POL model benefited the students’ CT concepts. The applied POL model was a process in which students actively constructed their understanding of programming concepts, as it focused on their understanding and application of CT concepts. The LAP learning model was a process by which teachers instilled programming concepts into students. Students passively accepted the programming concepts, so they did not understand the concepts well and were not proficient in the application. The application of the function to the two groups of students was not significant. Previous studies also found that some concepts are difficult for beginner programmers (Meerbaum-Salant et al., 2013). Grover et al. (2015) developed a course of “Foundations for Advancing Computational Thinking” to promote learners’ understanding of algorithmic concepts, but the mechanics of some constructs were difficult for learners to grasp in the context of text-based languages.

Although students can write and explain simple programs, they have difficulty with programs involving basic programming concepts (Brennan and Resnick, 2012). Students often struggle with algorithmic concepts, especially if teachers do not use appropriate supportive pedagogy to teach these concepts (Grover et al., 2015). In Python programming, the concept of functions includes built-in functions and custom functions. Built-in functions generally only require students to memorize and apply them, while custom functions require students to be able to build function modules by themselves. A function usually contains multiple CT concepts, and students need to be familiar with programming logic. For novice programmers, the application of functions is relatively limited, and the items involved in class are relatively simple. Students can directly write the corresponding code in the program without writing another function.

### Computational Thinking Practices

Computational thinking practice in this study included two aspects: “testing and debugging” and “reusing and remixing.” According to the results, the students in the POL mode had better CT practice than the students in the LAP learning mode in terms of “testing and debugging” and “reusing and mixing,” but there was no significant difference. In the POL model classroom, the teacher’s identity was more like a guide. Students designed possible problem solutions and tried them one by one. In this process, they continued to practice testing and debugging. Frequent operations helped develop their ability. In the LAP model classroom, the teacher explained the problem-solving solutions, and the students practiced on this basis, thereby avoiding many errors. Therefore, the students’ testing and debugging practices were lacking in this model.

Testing and debugging are indispensable for any type of problem solving (McCauley et al., 2008). Strict and systematic testing and debugging is an art and science in the field of computing, especially in the field of software development (Grover and Pea, 2013). However, the difference between the “testing and debugging” abilities of students in the two groups was not significant, which is consistent with previous studies. Fessakis et al. (2013) proposed that some students did not show any clear planning but rather tried commands one by one. For novice programmers, it is often difficult to link upper and lower command lines in groups (Robins et al., 2003), and they only analyze the single command line that includes a mistake (Lehrer et al., 1999). Hence, it is difficult to accurately and quickly find errors, correct them, and develop testing and debugging capabilities. Reusing and remixing involve comprehensive migration of problem solutions, and mastering skills in the original environment is essential for migration (Kurland and Pea, 1985). However, under the two learning models, the difference in the ability of students to “reuse and remix” was not significant. Previous research has shown that skill development usually requires sufficient training time (Bers et al., 2014; Atmatzidou and Demetriadis, 2016). On the other hand, most of the computer-based questions in this study were structured programming problems, and there was a lack of unstructured practical problems. Although this is conducive to the solution of the problem, it is not conducive to the development of migration capacity.

### Computational Thinking Perspectives

In this study, CT perspectives consist of “creation and expression,” “communication and cooperation,” “questioning,” and “problem solving.” The results showed that the CT perspectives of the students who adopted the POL model were better than those of the students who adopted the LAP model, and the difference was significant, indicating that the POL model was more effective in terms of cultivating students’ CT perspectives than the LAP learning model. Under the POL model, students expressed their own understanding of programming problems through programming to achieve self-creation and expression; in the process of finding solutions, students actively thought and discussed, and improved their communication and collaboration skills. When the solution was wrong or there was a conflict between their own thinking and the ideas of their classmates, the students would have doubts, so as to realize the optimal design of the algorithm. In the LAP model, teachers directly explained the process of problem-solving, while students were involved in the process of absorption, and there was less questioning and less interaction between students.

The result is consistent with previous research findings. Mouza et al. (2020) designed a 9-week after-school computational programming course, and collected long-term records of changes in students’ CT concepts, practices, and perspectives. Through interviews, it was found that the students’ CT perspectives greatly improved. Students were more willing to share programming works with classmates, which was a way to help them build confidence in programming. Moreover, repeated participation in computing courses made students use a number of computing perspectives. Burke (2012) found that middle school students could create their own digital stories through programming tools to express their CT perspectives. Kong and Wang (2020) also found that programming could improve CT perspectives. CT perspectives are connected to the formation of students’ thinking habits and personality, which have a significant influence on shaping teenagers’ cognition and values of the digital society (Deng et al., 2020). The CT perspectives require students to develop an understanding of themselves and relationships with others people and the technological world. When students express themselves in programming, CT perspectives are evident (Lye and Koh, 2014).

## Conclusion

This study constructed a POL model oriented to programming problems, and used a quasi-experiment to verify its effect on the cultivation of CT. In all, 60 eighth-grade students from a middle school in China were divided into an EG which adopted the POL model, and a CG which adopted the LAP learning model. The results showed that the students who were instructed with the POL model performed better than those who were instructed using the LAP model in terms of CT concepts, CT practices, and CT perspectives. Significant differences were found in CT concepts and CT perspectives, but no significant difference in CT practices.

### Implications

In terms of theory, although there have been studies on the relevance of CT and problem solving, there have been few empirical studies based on this theory. This study explored CT from the perspective of problem solving, and conducted empirical research, which not only enriches the related research on CT, but also provides theoretical references to explore CT in depth with a focus on problem solving. In terms of practice, this study provides a new practical perspective on how to cultivate the CT of middle school students, that is, relying on Python programming courses, applying the POL model, and imperceptibly cultivating students’ computational thinking. The study provides reference cases for computational thinking training and Python programming teaching, and provides an experience reference for teachers to carry out programming teaching.

### Limitations and Future Works

It should be noted that this study has some limitations. Firstly, the intervention time was short, just one semester. CT involves the use of computational science concepts and cognitive processes to solve problems creatively and efficiently (Anderson, 2016), and consists of multiple elements. Therefore, the cultivation of CT is not accomplished overnight. This study applied the POL model to a programming course. It is difficult to comprehensively and significantly improve students’ CT through only one semester of study. Further research can be conducted to investigate the effects of long-term use of this model.

Secondly, the fatigue response of the participants may have been a factor in the study. During the Python programming course, experimental participants needed to solve and complete a large number of programming problems and phased tests which could have led to their fatigue response in the later stage of the experiment. In addition, the assessment instrument modality may have had an impact on students’ performance (Atmatzidou and Demetriadis, 2016). CT can be applied in a wider learning environment instead of computational solutions (Kalelioğlu et al., 2016). Therefore, in future research, more CT evaluations that do not require a computer or programming platform should be developed (Tang et al., 2020).

## Data Availability Statement

The original contributions presented in the study are included in the article/supplementary material, further inquiries can be directed to the corresponding author.

## Author Contributions

All authors contributed equally to the conception of the idea, implementing and analyzing the experimental results, writing the manuscript, and reading and approving the final manuscript.

## Conflict of Interest

The authors declare that the research was conducted in the absence of any commercial or financial relationships that could be construed as a potential conflict of interest.

## Publisher’s Note

All claims expressed in this article are solely those of the authors and do not necessarily represent those of their affiliated organizations, or those of the publisher, the editors and the reviewers. Any product that may be evaluated in this article, or claim that may be made by its manufacturer, is not guaranteed or endorsed by the publisher.
